# Imaging human teeth by phosphorus magnetic resonance with nuclear Overhauser enhancement

**DOI:** 10.1038/srep30756

**Published:** 2016-08-08

**Authors:** Yi Sun, Ole Brauckmann, Donald R. Nixdorf, Arno Kentgens, Michael Garwood, Djaudat Idiyatullin, Arend Heerschap

**Affiliations:** 1 Radiology, Radboud University Nijmegen Medical Centre, Geert Grooteplein zuid 10, 6586 GA Nijmegen The Netherlands; 2 Solid State NMR, Institute of Molecules and Materials, Radboud University Nijmegen, Heyendaalseweg 135, 6525AJ Nijmegen, The Netherlands; 3 Division of TMD and Orofacial Pain Department of Diagnostic and Biological Sciences, University of Minnesota, 515 Delaware St. SE, Minneapolis, MN 55455, United States; 4 Center for Magnetic Resonance Research, University of Minnesota, 2021 Sixth Street SE, Minneapolis, MN 55455, United States

## Abstract

Three-dimensional phosphorus MR images (^31^P MRI) of teeth are obtained at a nominal resolution of 0.5 mm in less than 15 minutes using acquisition pulse sequences sensitive to ultra-short transversal relaxation times. The images directly reflect the spatially resolved phosphorus content of mineral tissue in dentin and enamel; they show a lack of signal from pulp tissue and reduced signal from de-mineralized carious lesions. We demonstrate for the first time that the signal in ^31^P MR images of mineralized tissue is enhanced by a ^1^H-^31^P nuclear Overhauser effect (NOE). Using teeth as a model for imaging mineralized human tissue, graded differences in signal enhancement are observed that correlate well with known mineral content. From solid-state NMR experiments we conclude that the NOE is facilitated by spin diffusion and that the NOE difference can be assigned to a higher water content and a different micro-structure of dentin. Thus, a novel method for imaging mineral content without ionizing radiation is proposed. This method has potential use in the assessment of de-mineralization states in humans, such as caries of teeth and osteoporosis of bones.

Common imaging tools to assess the mineral content in human tissues, such as bone and teeth, use ionizing radiation, which is associated with a health risk^1^. As the mineral component largely consists of calcium phosphates the imaging of phosphorus by MR represents a direct view on the mineral content in these tissues without ionizing radiation^2^,^3^,^4^,^5^. However, the application of ^31^P MR imaging to examine rigid structures such as bones and teeth is hampered by several factors affecting signal strength.

A major challenge in ^31^P MRI of bone and teeth is the rapid signal loss after excitation due to the ultra-short T_2_^*^ relaxation time of the ^31^P signals from mineral^5^. To avoid this loss the ^31^P signal must be acquired by MR pulse sequences with minimal delay between the excitation of spins and start of data sampling (‘dead time’ or ‘echo time’). Clinically relevant MRI sequences that accommodate minimal dead times are UTE (Ultra-short echo time)^6^,^7^, SWIFT (SWeep Imaging with Fourier Transformation)^8^ and ZTE (Zero Echo Time)^9^,^10^,^11^. The lower intrinsic MR sensitivity and slower longitudinal relaxation times of ^31^P spins compared to ^1^H spins are additional challenges to perform ^31^P MRI of bones and teeth. One way to enhance the ^31^P signal is by generating a ^1^H–^31^P nuclear Overhauser effect (NOE)^12^, from which also structural information can be obtained. The NOE has not been applied yet to MRI of mineralized tissue.

In this study we performed 3-dimensional ^31^P MRI with ^1^H-^31^P NOE to obtain anatomical and structural information of mineral tissue of human teeth as a model system with known phosphorous content in distinct anatomical regions. We adapted SWIFT and ZTE pulse sequences and the MR system hardware for NOE enhanced ^31^P MRI at a minimized ‘dead time’, and determined the structural origin of the NOE by solid-state NMR of mineral samples from teeth.

## Results

### Phosphorus MRI by ZTE and SWIFT pulse sequence

**Figure 1:**
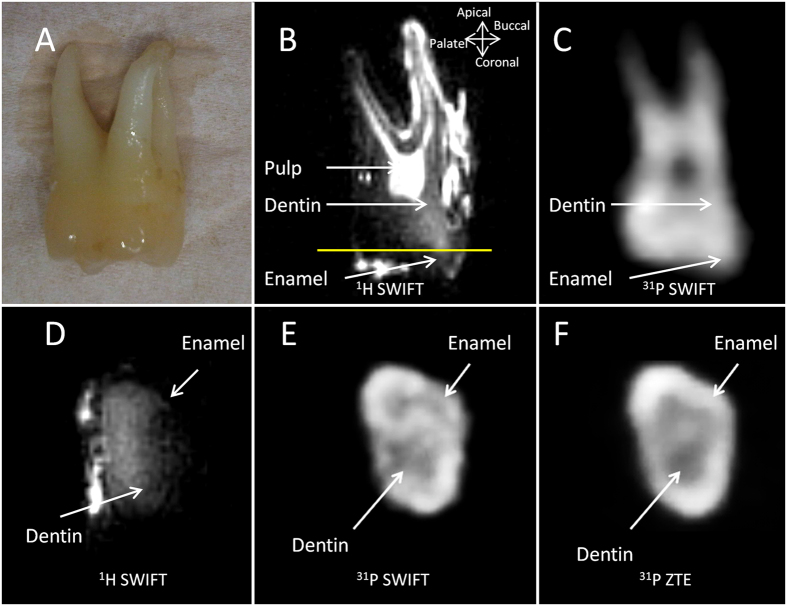
Photograph (**A**) and selected slices of 3D MR images of human maxillary left second molar tooth (**B**–**F**) including SWIFT ^1^H images (**B**,**D**), SWIFT ^31^P images (**C**,**E**) and ZTE ^31^P image (**F**) presenting axial (**B**,**C**) and apical (**D**–**F**) slices. The position of apical slices (**D**–**F**) is represented by the yellow line in image B. In the images of this slice, dentin and enamel are clearly separated. Image acquisition times were 100 s (**B**,**D**), 60 s (**C**) and 14  min (**E**,**F**).

For reference purposes, we first subjected a human maxillary left second molar tooth (Fig. 1A) to ^1^H MR imaging. In the images several anatomical features, such as dentin, enamel and pulp tissues can be distinguished (Fig. 1B). The hyperintense regions on the surfaces of the tooth are due to water present in the residual connective tissue attached to the mesiobuccal root, namely the periodontal ligament, and free water in the occlusal pits. From the ^1^H MR image in the apical-coronal direction, it is clear that enamel exhibits less intense signal than dentin (Fig. 1D).

In order to optimize the pulse sequence for ^31^P MRI of teeth, we measured the relaxation times of ^31^P nuclei: the global (whole tooth) T_2_^*^ of ^31^P was ≈100 μs and the global T_1_ was ≈130 seconds for this human maxillary left second molar tooth. For other teeth samples, similar T_2_^*^ values were obtained, and T_1_ values were all within the range of 100–200 seconds. In ^31^P MR images obtained in 100 seconds by SWIFT and ZTE (512 spokes), the contour of the molar tooth is already visible (Fig. 1C). In the higher resolution ^31^P MR image acquired in 14 minutes (4096 spokes, equal to a nominal resolution of approximately 0.5 mm)^6^, better delineation between structures with more details can be seen: for example, in cross-sectional slices enamel with higher phosphate content shows a higher signal intensity compared to dentin (Fig. 1E,F). The average signal-to-noise ratio (SNR) of the ^31^P signal of enamel is about 50% higher than that of dentin.

In general, the ^31^P MR images obtained with ZTE offer better SNR than those obtained with the gapped SWIFT sequence (compare Fig. 1E with 1F). The global SNR of the SWIFT images is 28% lower than the ZTE images (18.2 versus 25.3).

**Figure 2:**
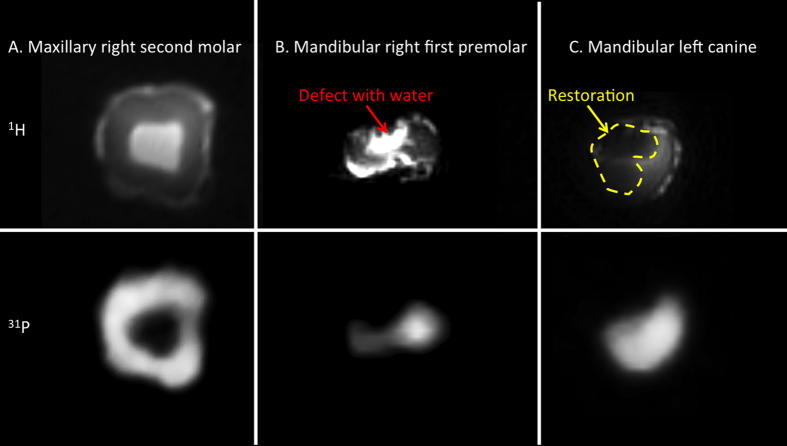
Comparison for ^1^H (top) and ^31^P (bottom) SWIFT MR images of three different teeth including human maxillary second molar (**A**), mandibular right first premolar (**B**) and mandibular left canine (**C**). The premolar contained a defect with high water content (red arrow); the left canine contains a restoration (yellow dashed curves).

For an investigation of the complementary anatomical information obtained by ^1^H MRI and ^31^P MRI we examined three differently affected teeth (Fig. 2). In the ^1^H MR image of a maxillary right second molar, the dentin and pulpal tissues are clearly separated: the pulpal tissues show much higher signal intensity compared to the dentin. In contrast, the ^31^P MR image shows a void for the pulpal tissue, due to a much lower phosphate content (mainly phospholipids in nerves, vessels, etc.), and dentin is visible as a hyperintense region (Fig. 2A). In the ^1^H MR images of a mandibular right first premolar (Fig. 2B), the buccal pit and distolingual carious lesions in the crown are clearly visible because of the increased water content in these lesions (Fig. 2B top). Complementary, the ^31^P MR image identifies mineral breakdown in this area (Fig. 2B bottom). For the maxillary left canine, a composite restoration at the mesiolingual surface of the tooth is responsible for the hypointense region (dashed curves in yellow dashed curve) observed on the ^1^H MR image (Fig. 2C top). The ^31^P MR image better represents the structure of the canine by showing its mineral content (Fig. 2C bottom).

### NOE enhancement in ^31^P MRI

**Figure 3:**
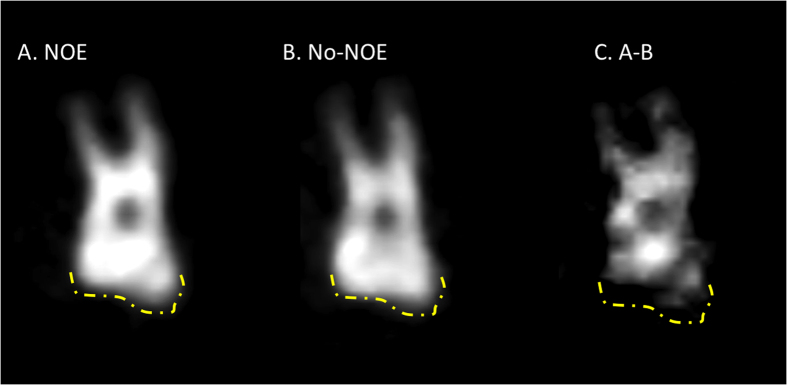
Selected slices of 3D ^31^P MR images of maxillary left second molar tooth obtained with the SWIFT sequence. (**A**) SWIFT image obtained with irradiation at the ^1^H frequency for 1.282 s, preceded by 64 dummy scans to reach steady state. (**B**) MR image of same slice obtained without ^1^H irradiation. (**C**) The subtraction of A and B shows a positive NOE in particular for signal coming from dentin. The yellow dash-dot line represents the contour of the enamel.

Irradiation at the ^1^H frequency generates a positive NOE for the ^31^P MR signal of teeth (Fig. 3). The global NOE enhancement in the ^31^P MR images is around 20% (an SNR of 21.8 in SWIFT MR images with NOE versus 18.2 without NOE, measured over the whole object). This enhancement is not the same for all regions: subtraction of the SWIFT MR images with and without NOE clearly reflects the shape of dentin, while enamel is less intense in the subtraction image (Fig. 3C). The estimated NOE enhancement for dentin and enamel from these images is about 20% and 4% respectively.

### Solid-state spectroscopic studies of ^1^H-^31^P NOE of teeth

**Figure 4:**
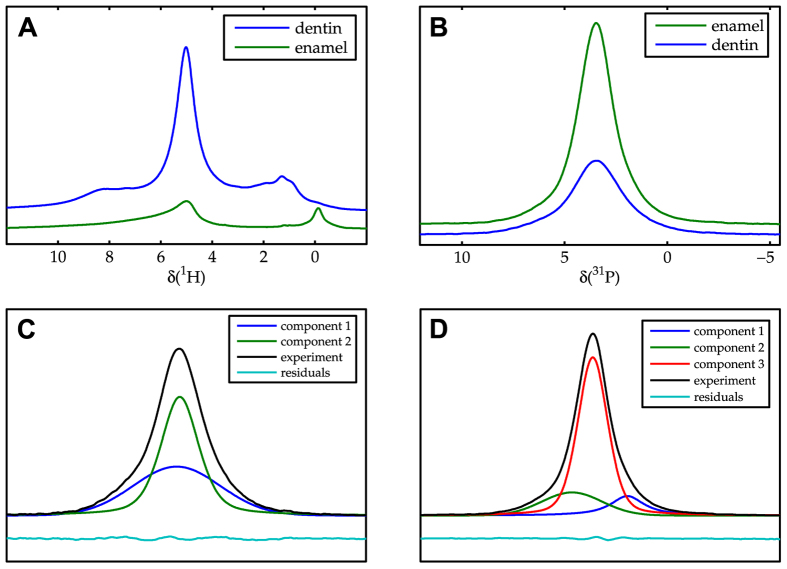
^1^H MAS spectra (ν_r_  = 35 kHz, **A**) and ^31^P MAS spectra at (ν_r_ = 10 kHz, **B**) of dentin (blue lines) and enamel (green lines), the spectra are recorded with the same parameters and processed in the same way. The height of the spectrum is scaled normalized to the sample weight, allowing for a semi-quantitative judgment. Deconvolution of the dentin ^31^P signal (**C**) and the enamel ^31^P signal (**D**) using two and three components^12^,^13^.

To investigate the origin of the ^1^H-^31^P NOE enhancements in teeth, we performed solid-state NMR spectroscopy on separated dentin and enamel materials. In ^1^H magic angle spinning (MAS) NMR spectra of both enamel and dentin, the water resonance at ≈5 ppm is the most pronounced signal (Fig. 4A). In enamel, a characteristic resonance for the hydroxyl group in hydroxyapatite arises at around ~0.1 ppm, while no signal can be seen at this chemical shift for dentin (Fig. 4A). The other resonances in dentin arise from the organic matrix^13^,^14^. The ^31^P MAS spectrum shows lower signal intensity for dentin compared to enamel (Fig. 4B). The ^31^P spectrum of dentin and enamel were deconvoluted based on previous studies (Fig. 4C,D). For dentin, two components are assigned for a crystalline and an amorphous phase^15^. For enamel, a three-component model is used. The central line is assigned to interior phosphate groups. The shoulders have been assigned to unprotonated and protonated phosphate surface groups^16^. The different components constituting the phosphorus spectrum behave similarly in the NOE build-up.

**Figure 5:**
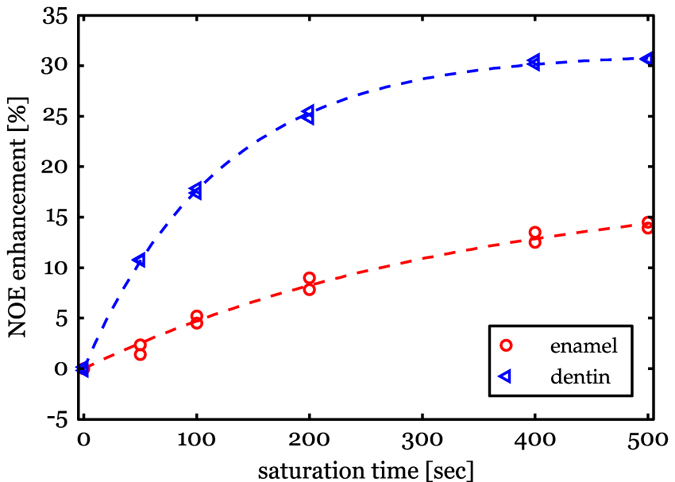
Measured NOE build-up of the ^31^P signal of dentin (blue) and of enamel (red) as a function of ^1^H saturation time. The dashed lines represent mono-exponential fits to the data points.

The ^1^H-^31^P NOE enhancement as a function of the saturation time can be fitted by mono-exponential functions and shows a clear difference in the NOE build-up for dentin and enamel (Fig. 5). At the longest used ^1^H irradiation time of 500 s the NOE plateaus at 31% for dentin, but for enamel this plateau is not yet reached with an enhancement of 14%. Extrapolation of the build-up curve for enamel indicates a plateau at 19%. The NOE build-up rate in dentin is faster than in enamel with rate constants of about 116 s and 310 s respectively (Fig. 5).

**Figure 6:**
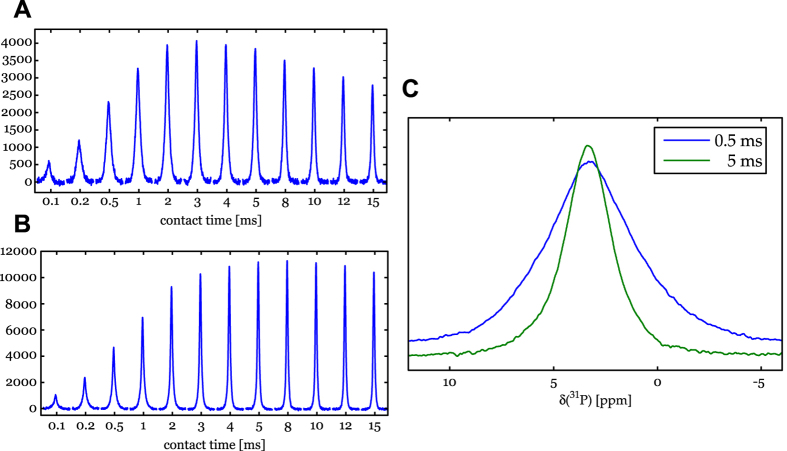
Array of CP spectra at different contact times for dentin (**A**) and enamel (**B**). Comparison of the line shapes of the ^1^H → ^31^P CP spectra of dentin at short and long contact times (**C**).

The results of ^1^H → ^31^P cross-polarization (CP) experiments are presented in Fig. 6. The dentin polarization reaches a maximum at ≈3 ms CP contact time; then this polarization decreases and the signal intensity further decays at longer contact times (Fig. 6A). Enamel polarizes slower: the build-up of the CP signal intensity only reaches its maximum intensity after 8 ms; and there is no prominent decrease even until 15 ms (Fig. 6B). A comparison of the ^1^H → ^31^P CP spectra of dentin at short and long contact times shows that the ^31^P line shape of dentin observed at long CP contact times is narrower (Fig. 6C) and similar to the line shape observed in the ^31^P and NOE enhanced ^31^P spectra.

## Discussion and Conclusion

In this study we realized 3-dimensional ^31^P MR imaging of human teeth down to nominal resolutions of about 0.5 mm in measurement times below 15 minutes showing resolved dentin and enamel structures. In the images of diseased teeth spatially reduced phosphate contents were observed complementary to lesions seen in ^1^H MR images obtained with the same probe. The ^31^P MR signal in teeth can be enhanced by the ^1^H-^31^P NOE, which also provides an option for additional imaging contrast as the NOE is different for dentin and enamel. Solid state NMR revealed that the origin of this difference in NOE is the higher water content and different microstructure of dentin.

It is attractive to use ^31^P MR imaging for direct spatial information on the mineral content of teeth and bone as the major component is hydroxylapatite (Ca_10_(PO_4_)_6_(OH)_2_)^17^. However, MRI of ^31^P in human mineralized tissue is challenging, owing to its low MR sensitivity and very long T_1_ and short T_2_^*^ relaxations. To deal with the short signal persistence after excitation we implemented hardware for rapid switching between excitation and receive mode and ZTE and SWIFT sequences for immediate signal acquisition. The SNR of SWIFT images is expected to be lower compared to images obtained by ZTE due to gapped acquisition and is proportional to the square root receiver duty cycle d_rec_(=0.5 in this study). This is in agreement with experimental findings: 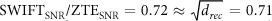. However, SWIFT requires less RF peak power, which is an advantage over ZTE for *in vivo* imaging. A recent modification of SWIFT, multi-band SWIFT, allows for a highly increased receiver duty cycle^18^. Both methods, ZTE and SWIFT, are becoming more widely available and have potential for ^31^P MRI of human bone and teeth. Various ^31^P MRI methods have been used to assess mineralization of bone, including a solid-state method similar to ZTE for human teeth^19^,^20^,^21^. In the present study we obtained a higher resolution, clearly resolving dentin and enamel, at shorter acquisition times.

The NOE is the fractional change in the integrated NMR absorption intensity of a nuclear spin system upon saturation of neighbor-coupled spins^22^. ^31^P NOE enhancement by ^1^H irradiation has been widely reported for phosphate compounds in solutions^23^,^24^, but never in human mineralized tissue. In this study, a positive ^1^H-^31^P NOE enhancement of the signal intensity in ^31^P MR images is observed. More importantly, the enhancement for enamel and dentin is different. Steady state NOE experiments show that the enhancement reaches its maximum value with ^1^H RF irradiation lasting about 5 × T_1_ of ^31^P spins, in agreement with our knowledge of heteronuclear NOE in the solid state^25^.

NOE and CP both rely on the dipolar interaction. As the phosphate structures in enamel and dentin are chemically very similar no big changes in the static dipolar interactions are expected. However, molecular motions will modulate the dipolar interactions and therefore affect CP and NOE efficiency. Different microstructures of chemically related moieties can therefore still give different CP and NOE response. The difference in microstructure of enamel and dentin is clearly reflected in the CP dynamics. CP is a coherent polarization transfer process and is very sensitive to motions in the kHz regime of the involved spins. The slower build-up in enamel most likely results from much lower relative ^1^H content in enamel compared to dentin. The change in line-width in the ^31^P CP spectra at longer contact times indicates that the materials consist of various components showing different dynamics. In dentin, the broader line-width at short contact times is attributed to a more heterogeneous, amorphous environment^26^,^27^,^28^.

To identify differences in NOE build-up for the various components of enamel and dentin, the NOE enhanced ^31^P spectra were deconvoluted according to the number of components used in literature^14^,^15^. However, within the error of the analysis, all individual components in enamel and dentin show similar enhancement and build-up dynamics. We attribute this to ^31^P spin diffusion (neighboring spins exchange polarization via dipolar couplings) taking place during the build-up process over hundreds of seconds. The NOE effect is a cross-relaxation effect, which requires a modulation of the dipolar interaction at the nuclear Larmor-frequency to be efficient. Therefore fast motions with correlation times in the ns regime must be present. The OH^−^ concentration in enamel is much higher than in dentin, but the strongest enhancement is observed in dentin. This indicates that the hydroxyl resonance is not the main contributor to the NOE mechanism. As the water resonance around 5 ppm is a common feature and remains even after extensive drying^29^, water appears to be strongly adsorbed to the minerals. This is in agreement with the observation that the NOE persists after drying. The water resonance is more pronounced in dentin, which is consistent with the higher enhancement observed, and is therefore the most plausible source of the NOE. The structural role of water in bio-minerals is still under investigation^30^.

We tried to identify the ^1^H resonance involved in the NOE by ^1^H saturation at selective frequencies, but no significant differences in the enhancement were observed (data not shown). It is virtually impossible to selectively saturate a single proton resonance as during the long NOE build-up period (hundreds of seconds), irradiating at a single frequency, spin diffusion will effectively equilibrate all polarization. This is in agreement with experiments on synthetic apatite, where all proton resonances show spin exchange within milliseconds^31^. Since the NOE enhanced ^31^P spectra of dentin resemble it’s CP spectra at long polarization times (reflecting the crystalline phase remote from protons), it is most probable that the more pronounced and faster NOE build-up in dentin arises in the amorphous phase and is then transferred by spin diffusion to the crystalline phase.

A difference in dentin and enamel microstructure most likely explains the faster and more pronounced NOE in dentin: enamel consists mainly of highly crystalline apatite mineral (96 wt%), while dentin, besides being partly amorphous, also contains an organic matrix and water (i.e. type I collagen fibrils at around 20 wt% and hydration/interstitial water 10 wt%)^32^. High frequency motions modulating the ^1^H-^31^P dipolar interactions are most likely to occur in these phases.

Compared to 3D imaging with cone-beam computed tomography, ^31^P MRI uses no ionizing radiation and it can be combined with ^1^H MRI for the visualization of calcified dental tissue. Additionally ^31^P MRI has an advantage of being a true volumetric method. Although the current results suggest some favorable prospects for biomedical applications, the realization of ^31^P MRI for human bone imaging to address clinical questions faces a number of challenges^33^. In this respect it is of interest that the RF duty cycle for ^1^H irradiation in the current NOE experiments was less than 5%, while much higher duty cycles for ^1^H-^31^P NOE of the human brain have been safely applied at 7T field strength^34^.

In conclusion, we demonstrate that 3D ^31^P MR images of teeth can be obtained by SWIFT and ZTE pulse sequences with minimum transmit/receive deadtime, in which dentin and enamel are spatially resolved. We showed that irradiation at the ^1^H frequency creates NOE enhancement of the ^31^P signal in these images. The signal of dentin is more enhanced than that of enamel which is caused by micro-structural differences. Thus the NOE provides an option for additional structural imaging of teeth. This new approach may also have potential in the assessment of other mineralized tissues in the human body, such as monitoring mineral changes due to osteoporosis.

## Materials and Methods

### Extracted human teeth samples

Extracted teeth harvested as waste tissue were selected for *ex-vivo* imaging. No data that could identify patients were recorded and therefore was Institutional Review Board exempt. Teeth were stored in an isotonic saline solution containing 0.1% of sodium azide as an antimicrobial agent. Before the MRI experiments, water on the surface of the teeth was removed by blotting with paper towels, and teeth were then wrapped with Teflon tape to reduce evaporation of water and isolate the tissue from equipment. A maxillary left second molar and a mandibular right second molar were chosen to test the feasibility of ^31^P MRI to access their overall anatomy and mineral content. In order to further test whether ^31^P MRI can detect mineral loss, two teeth with carious defects were selected: a mandibular right first premolar with buccal pit lesion and distolingual lesion in the crown; a maxillary left canine with restoration at the mesiolingual surface.

### MR hardware and pulse sequences

MRI was performed on a 9.4T MR system (Agilent-Varian) with a home-built double resonance (^31^P and ^1^H) probe. The phosphorus RF coil consisted of two parallel copper loops of 1.5 cm in diameter at 1 cm distance. The proton RF coil was a copper single loop surface coil with 1.5 cm in diameter positioned in between the phosphate coil loops and oriented perpendicularly to them. The RF hardware allowed minimum “dead” times between excitation and acquisition and was 2 μs for the ^1^H frequency and 3 μs for the ^31^P frequency.

**Figure 7:**
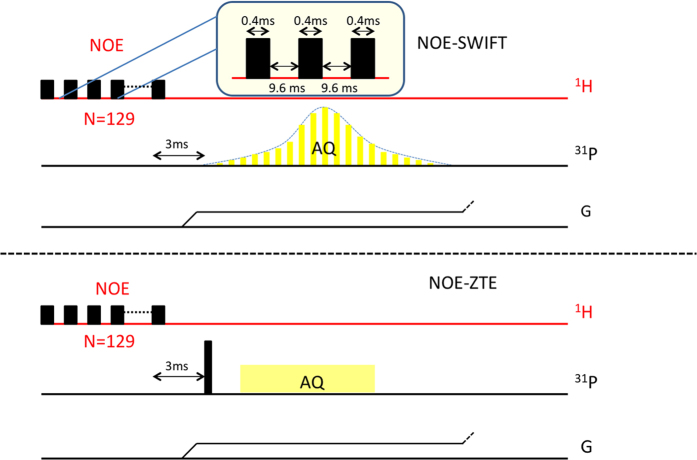
Schematic representation of SWIFT and ZTE pulse sequences with saturation pulses applied at the proton frequency. For SWIFT, the excitation pulse is divided into segments, each switching RF power on and off for signal acquisition (yellow colored). For both pulse sequences, the gradients were set to the required direction and amplitude. They remained constant until adjusted for the next repetition. The saturation pulse train comprises of 129 blocks of 10 ms each with a hard pulse of 400 μs width with a delay of 9.6 ms in between.

The SWIFT pulse sequence^35^,^36^ consists of a series of excitation RF pulses and simultaneous data acquisition (sampled in the gaps) in the presence of a readout gradient (Fig. 7). For the excitation pulse, an amplitude- and frequency-modulated hyperbolic secant based (HS2) pulse was used^37^,^38^,^39^. The phases of sequentially excited signals were unscrambled by correlation with the pulse function in the frequency domain. The ZTE sequence^40^,^41^ comprised of a short hard RF pulse with the readout gradient already on, followed by the acquisition (Fig. 7). Both sequences used small incremental changes of gradients between projections. The images were reconstructed from the acquired data using a gridding algorithm.

### MRI experiments

Proton SWIFT images were acquired with an excitation bandwidth of 125 kHz, 256 gaps for sampling, an oversampling factor of 4, and 15° flip angle (FA). The field of view (FOV) was 3 × 3 × 3 cm. In total 32,000 radial spokes were acquired with a repetition time (TR) of 2.5 ms which includes a 2 ms total pulse length. The total acquisition time (TA) was 100 seconds. Images were reconstructed to a matrix of 256 × 256 × 256 pixels. Phosphorus SWIFT and ZTE images were both acquired with an excitation bandwidth of 62.5 kHz, 64 gaps for sampling, FA of 3.7°, oversampling factor of 4, TR of 200 ms, FOV of 3 × 3 × 3 cm^3^, TA of about 100 s for a low resolution (512 spokes) or 14 minutes for a higher resolution image (4,096 spokes), and receiver duty cycle (d_rec_) of 0.5 in all the experiments. Almost all other parameters are similar, except for the short hard pulse excitation (5 μs length) applied in ZTE. Images were reconstructed to a matrix of 64 × 64 × 64 pixels. The global T_1_ and T_2_^*^ relaxation times of the ^31^P spins were estimated (for the whole tooth) by inversion recovery and measuring line widths, respectively. Two regions of interests (ROIs) localized on dentin and enamel were manually drawn on the cross-sectional slices of the ^31^P images, to compare their difference in SNR. The SNR is evaluated by calculating the average signal divided by the standard deviation of the background signal.

### Nuclear Overhauser effect enhanced ^31^P MR imaging

For the ^1^H-^31^P NOE enhanced experiments, the radiofrequency saturation of proton spins was implemented in the steady-state imaging scheme. The proton spins were saturated for 1.282 s during TR (1.482 s) by a hard pulse train (Fig. 7) of 129 hard pulses being 400 μs long with a pulse delay of 9.6 ms, by which full saturation of the proton magnetization was achieved^42^. This preparation pulse train for the NOE was the same for both ZTE- and SWIFT-images. The delay between the last ^1^H saturation pulse and the ^31^P excitation pulses was also the same and equal to 3 ms. To reach steady state for the NOE we applied 64 dummy scans in front of the MR acquisition. The saturation pulses were turned off for the non-NOE enhanced experiment, but the TR remained unchanged. For the estimation of the extent of the NOE the TA for ^31^P MRI both with and without NOE was about 48 minutes.

### Solid-State NMR

To quantify the NOE build-up and to identify the source of the enhancement, a series of solid-state NMR spectroscopy experiments were performed. Dentin and enamel were separated and crushed into powder. Single pulse ^1^H NMR spectra were recorded on a Varian VNMRS 20 T solid-state NMR spectrometer using a 1.6 mm probe head under MAS at 35 kHz with a pulse length of 2.9 μs and relaxation delay of 5 seconds. ^31^P single pulse excitation and NOE experiments were performed on a 9.4T solid-state NMR system (Agilent-Varian), with a 3.2 mm rotor and a H-X-Y triple resonance probe head using a relaxation delay of 750 s at 10 kHz spinning rate. The ^31^P excitation pulse length was 5.5 μs. The ^1^H nuclei were saturated by a train of 1 ms soft pulses (ν_1_ = 450 Hz) separated by a delay of 1 ms, with a total saturation time ranging from 50 to 500 seconds. ^1^H-^31^P CP spectra at different contact times (100 μs − 15 ms) were recorded for dentin and enamel at 9.4T with a recycle delay of 10 s, an ^1^H excitation pulse length of 5.4 μs and 10 kHz spinning.

## Additional Information

**How to cite this article**: Sun, Y. *et al.* Imaging human teeth by phosphorus magnetic resonance with nuclear Overhauser enhancement. *Sci. Rep.* **6**, 30756; doi: 10.1038/srep30756 (2016).
